# Microbial enzymes for the recycling of recalcitrant petroleum‐based plastics: how far are we?

**DOI:** 10.1111/1751-7915.12710

**Published:** 2017-03-28

**Authors:** Ren Wei, Wolfgang Zimmermann

**Affiliations:** ^1^ Department of Microbiology and Bioprocess Technology Institute of Biochemistry Leipzig University Johannisallee 21‐23 04103 Leipzig Germany

## Abstract

Petroleum‐based plastics have replaced many natural materials in their former applications. With their excellent properties, they have found widespread uses in almost every area of human life. However, the high recalcitrance of many synthetic plastics results in their long persistence in the environment, and the growing amount of plastic waste ending up in landfills and in the oceans has become a global concern. In recent years, a number of microbial enzymes capable of modifying or degrading recalcitrant synthetic polymers have been identified. They are emerging as candidates for the development of biocatalytic plastic recycling processes, by which valuable raw materials can be recovered in an environmentally sustainable way. This review is focused on microbial biocatalysts involved in the degradation of the synthetic plastics polyethylene, polystyrene, polyurethane and polyethylene terephthalate (PET). Recent progress in the application of polyester hydrolases for the recovery of PET building blocks and challenges for the application of these enzymes in alternative plastic waste recycling processes will be discussed.

## Introduction

**Figure 1:**
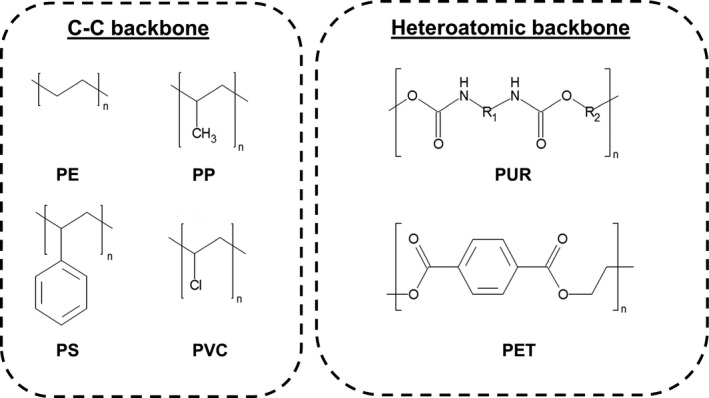
Backbone structural formula of widely used petroleum‐based plastics (PlasticsEurope, 2016).

Plastics can be easily moulded into different shapes and forms (Andrady, 2015b,d). Due to their low weight, durability and low production cost, they can be readily manufactured to an expanding range of products used for a variety of civil and industrial applications (Andrady and Neal, 2009; Thompson *et al*., 2009; Andrady, 2015d). In many areas, they have substituted natural materials as well as paper and glass in most of their former uses (Andrady and Neal, 2009). As a result, plastics have become omnipresent in our daily life. Over the last 50 years, the global production of plastics has continuously increased and reached 322 million tons in 2015 (PlasticsEurope, 2016). In Europe, packaging (39.9%), building and construction (19.7%) and automotive (8.9%) are the leading application sectors for the plastic industry (PlasticsEurope, 2016). The majority of plastics are made from fossil‐based feedstocks (Hopewell *et al*., 2009; Andrady, 2015d). Polyethylene (PE), polypropylene (PP), polystyrene (PS), polyvinyl chloride (PVC), polyethylene terephthalate (PET) and polyurethane (PUR) are the main types of plastics, which correspond to over 80% of the total demand in Europe (PlasticsEurope, 2016; Fig. 1). It has been estimated that nearly 8% of the total global fossil fuel production is utilized as raw materials or to provide energy for the manufacturing of plastics (Hopewell *et al*., 2009; Andrady, 2015d).

As a result of the widespread use and consumption of plastic products, up to 25.8 million tons of post‐consumer plastic wastes is annually generated in Europe alone (PlasticsEurope, 2016). Packaging materials or other short‐lived disposable plastic items that are discarded within a year of manufacturing make up nearly 60% of the total plastic waste in Europe (WRAP, 2016). In 2014, 69% of post‐consumer plastic waste in Europe was recovered by recycling and energy generation processes, whereas 31% still ended up in landfills (PlasticsEurope, 2016).

Most of the petroleum‐based plastics have been considered as notably resistant to microbial degradation (Andrady, 1994; Zheng *et al*., 2005; Mueller, 2006; Tokiwa *et al*., 2009). The majority of plastics manufactured today are therefore estimated to persist in the environment for a very long time (Hopewell *et al*., 2009; Andrady, 2015a). In addition, the careless disposal of plastics waste, especially in developing countries, is aggravating the associated environmental problems (Andrady, 1994, 2015a; Rigamonti *et al*., 2014; Jambeck *et al*., 2015). The degradation process of plastic waste causing serious environmental damages can be expected to be considerably different in landfills, terrestrial and marine environments (Kyrikou and Briassoulis, 2007; Andrady, 2015a; Gewert *et al*., 2015). It has been shown that hazardous chemicals released from plastic waste in landfills can contaminate the groundwater (North and Halden, 2013). An increasing amount of plastic waste is also entering marine environments. A calculated up to 12 million tons of plastics produced by coastal countries worldwide has entered the oceans in 2010 and this amount is expected to grow steadily (Jambeck *et al*., 2015). Plastic pollution has deadly effects on marine mammals from ingestion or getting entangled in plastic debris (Derraik, 2002; Andrady, 2015e; Wilcox *et al*., 2015; Nelms *et al*., 2016). Microplastics, partially degraded plastic debris of less than 5 mm in diameter, have been shown to pose an even more serious impact on marine ecosystems by concentrating persistent organic pollutants. These are often hydrophobic compounds with a high affinity to microplastics, thereby entering the food chains when microplastics are ingested by marine wildlife (Andrady, 2011, 2015e; Van Cauwenberghe *et al*., 2013; Law and Thompson, 2014).

The development of biodegradable plastics is providing a promising alternative to their counterparts made from petrochemicals (Andrady, 2015d; Iwata, 2015). Due to their lower durability and lack of compatibility with existing equipment and end‐of‐life management systems, the scale of production and use of biodegradable plastics are, however, still very limited (Gourmelon, 2015). In addition, not all of the so‐called bioplastics derived from renewable resources are readily biodegradable (Tokiwa *et al*., 2009; Soroudi and Jakubowicz, 2013; Yates and Barlow, 2013; Andrady, 2015d; Prieto, 2016). They may also persist in the environment for a considerable long time depending on local abiotic factors that facilitate their breakdown and subsequent biodegradation (Swift, 2015; Hopewell *et al*., 2009; Andrady, 2015a). The enzymatic degradation of biodegradable plastics has been reviewed elsewhere (Tokiwa *et al*., 2009; Bhardwaj *et al*., 2013; Banerjee *et al*., 2014).

**Figure 2:**
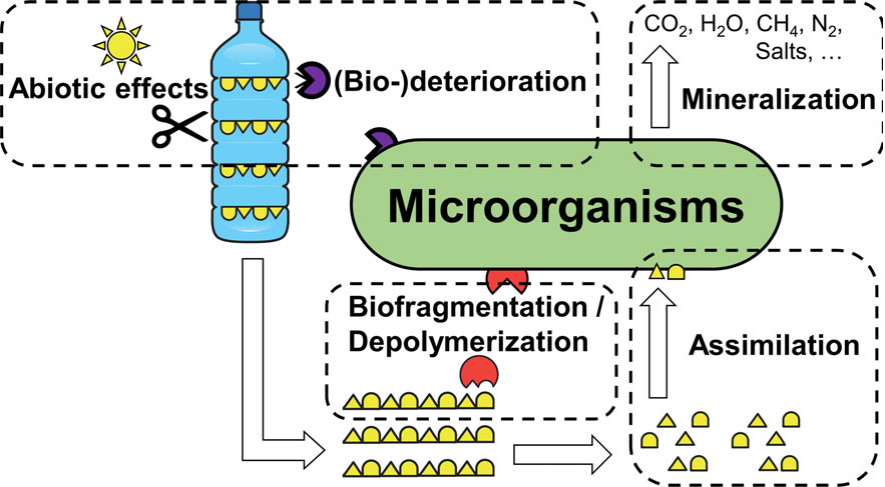
Schematic illustration of plastic biodegradation (Lucas *et al*., 2008).

The biodegradation of recalcitrant plastics has become a focus of research (for recent reviews, see Eubeler *et al*., 2010; Gu, 2003; Krueger *et al*., 2015; Lucas *et al*., 2008; Shah *et al*., 2008b; Sivan, 2011; Zheng *et al*., 2005). The complex process of their biodegradation in the environment has been considered as the result of a combination of many abiotic and biotic factors (Mueller, 2006; Lucas *et al*., 2008; Sivan, 2011). As schematically shown in Fig. 2, following a deterioration cooperatively accomplished by abiotic factors and microorganisms, the bulk polymer becomes fragmented with more exposed surfaces available for biological attack. Inducible extracellular enzymes play a crucial role in the further depolymerization process of the synthetic polymers (Lucas *et al*., 2008; Sivan, 2011; Bhardwaj *et al*., 2013). Plant polymers are the natural substrates for key enzymes capable of attacking the polymer backbones of synthetic plastics. For example, cutinases can hydrolyse cutin, an aliphatic polyester found in the plant cuticle (Kolattukudy, 1981; Heredia, 2003). These enzymes are also able to hydrolyse the ester bonds in PET and PUR (Chen *et al*., 2013; Wei *et al*., 2014c; Schmidt *et al*., 2017). Several enzymes involved in the metabolism of plant lignin are also involved in the degradation of the thermoplastic polyolefin PE (Sivan, 2011; Restrepo‐Flórez *et al*., 2014).

Once the molecular size of the synthetic polymers has been reduced to a range of 10–50 carbon atoms, the degradation products can be taken up into the cell for further metabolization (Lucas *et al*., 2008; Restrepo‐Flórez *et al*., 2014). The intracellular enzymatic processes involved are beyond the scope of this review.

We will briefly summarize recent advances in the enzymatic degradation of the widely used recalcitrant petroleum‐based plastics PE, PS, PUR and PET and discuss the challenges for the development of efficient plastic‐degrading enzymes and their potential use in alternative recycling processes.

## Physiochemical properties of synthetic plastics as obstacles for their enzymatic degradation

**Figure 3:**
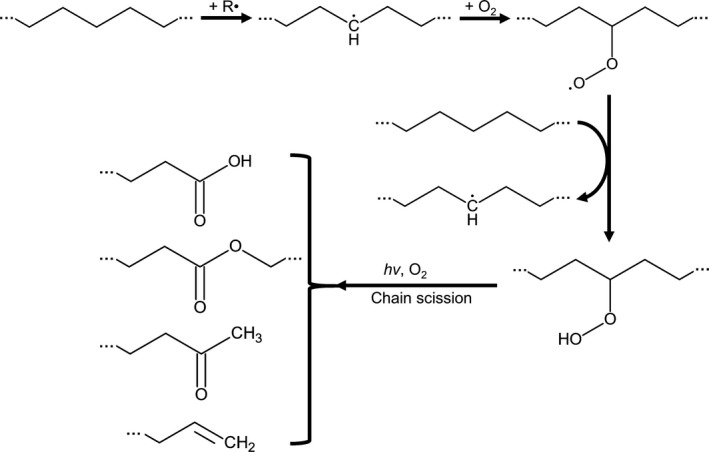
Simplified scheme of the abiotic degradation of polyethylene by oxygen and light. Radicals (R^·^) can be generated by photo‐oxidation mediated by chemical oxidants (modified based on Koutny *et al*., 2006b).

Synthetic plastics show a high resistance to many physical, chemical and biological factors (Andrady and Neal, 2009; Thompson *et al*., 2009). However, this durability also results in their extremely slow degradation in the environment. The hydrophobicity, degree of crystallinity, surface topography and molecular size of the synthetic polymers are important factors restricting their biodegradability (Tokiwa *et al*., 2009; Webb *et al*., 2013; Restrepo‐Flórez *et al*., 2014). Polymers with hydrolysable chemical bonds in their backbone such as PET (Webb *et al*., 2013) and PUR (Cregut *et al*., 2013) are more susceptible to biodegradation than PE, PS, PP and PVC (Zheng *et al*., 2005; Tokiwa *et al*., 2009; Krueger *et al*., 2015; Fig. 1). Their highly stable carbon‐carbon (C‐C) bonds have to be oxidized first prior to their further depolymerization (Zheng *et al*., 2005; Restrepo‐Flórez *et al*., 2014). Abiotic factors such as UV irradiation, oxygen, temperature, as well as the presence of chemical oxidants, therefore play a crucial role in the degradation of PE and PP in the environment (Bonhomme *et al*., 2003; Jakubowicz, 2003; Hakkarainen and Albertsson, 2004; Koutny *et al*., 2006a,b; Arkatkar *et al*., 2010; Fig. 3).

The high molecular weight of synthetic polymers with hydrophobic repeating units determines their insolubility in water prohibiting a rapid assimilation by microorganisms (Zheng *et al*., 2005; Arutchelvi *et al*., 2008; Restrepo‐Flórez *et al*., 2014; Krueger *et al*., 2015). Their degradation by enzymes can be considered as a surface erosion process that is strictly depending on the surface properties of the polymers (Mueller, 2006; Lucas *et al*., 2008; Restrepo‐Flórez *et al*., 2014). A high degree of hydrophobicity, a low specific surface area and a smooth surface topography restrict the formation of a biofilm by polymer‐degrading microorganisms (Lucas *et al*., 2008; Loredo‐Trevino *et al*., 2012; Cregut *et al*., 2013; Restrepo‐Flórez *et al*., 2014; Wei *et al*., 2014a). The hydrophobic polymer surface has also been shown to prohibit an effective adsorption and catalytic performance of polymer‐degrading enzymes (Espino‐Rammer *et al*., 2013; Ribitsch *et al*., 2015, 2013; Sammond *et al*., 2014). The microbial degradation is furthermore restricted by the low surface‐to‐volume ratio of the plastic debris. A micronization of different PET materials to obtain particle sizes between 0.25 and 0.5 mm was shown to markedly improve their subsequent degradation by a bacterial polyester hydrolase by increasing the accessible surface area for the enzyme (Gamerith *et al*., 2017). A pretreatment of plastic waste may therefore be a prerequisite in biocatalytic recycling, thereby resulting in further process costs.

**Figure 4:**
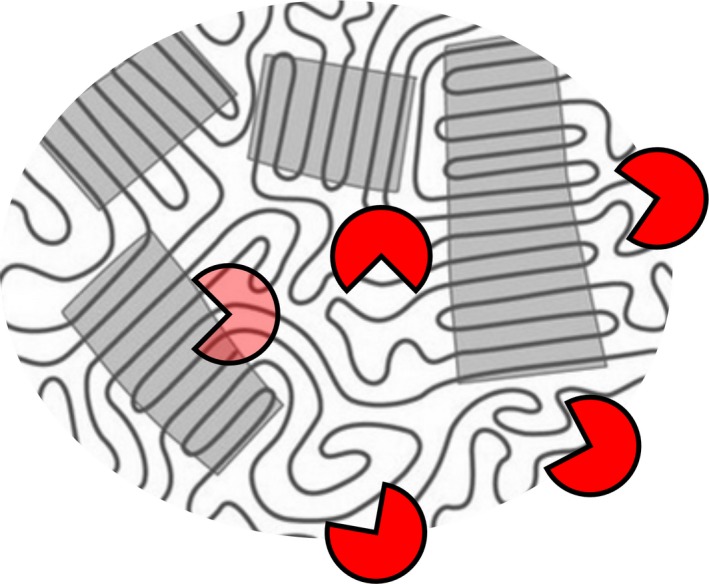
Schematic illustration of a semi‐crystalline polymer containing both amorphous and crystalline regions (grey areas). The amorphous parts are more susceptible to enzymatic attacks.

Most petroleum‐based plastics are semi‐crystalline polymers containing both crystalline and amorphous regions, the latter being more susceptible to microbial attacks (Sarkar *et al*., 2006; Tokiwa *et al*., 2009; Loredo‐Trevino *et al*., 2012; Webb *et al*., 2013; Restrepo‐Flórez *et al*., 2014; Fig. 4). The degree of crystallinity of the polymers has therefore a strong influence on their biodegradability (Urgun‐Demirtas *et al*., 2007; Brueckner *et al*., 2008; Jenkins and Harrison, 2008; Eberl *et al*., 2009; Ronkvist *et al*., 2009; Horn *et al*., 2012; Webb *et al*., 2013; Restrepo‐Flórez *et al*., 2014; Wei *et al*., 2014a). However, the crystalline parts of synthetic plastics can also be degraded enzymatically. For example, the crystalline fraction of a thermally pretreated PE sample was also degraded following the complete consumption of the amorphous parts (Manzur *et al*., 1997; Restrepo‐Flórez *et al*., 2014). The complete decomposition of low crystalline PET films by a fungal polyester hydrolase has been shown to occur at an almost linear rate, indicating that the crystalline parts were also attacked by the enzyme (Ronkvist *et al*., 2009).

## Enzymatic degradation of plastics with carbon‐carbon backbones

Biodegradation of PE, PP, PS and PVC is hampered by the lack of hydrolysable functional groups in their backbones (Tokiwa *et al*., 2009; Restrepo‐Flórez *et al*., 2014; Krueger *et al*., 2015). The initial breakdown of the polymers in the environment and the observed reduction in their molecular weight have been mainly attributed to a synergistic action of biotic and abiotic factors (Bonhomme *et al*., 2003; Hakkarainen and Albertsson, 2004; Eubeler *et al*., 2010; Restrepo‐Flórez *et al*., 2014). The carbonyl groups formed as a result of UV irradiation or oxidizing agents have been considered as more accessible for a subsequent microbial attack (Albertsson *et al*., 1987; Karlsson *et al*., 1988; Albertsson and Karlsson, 1990; Koutny *et al*., 2006a,b; Fontanella *et al*., 2010; Fig. 3). Biodegradation studies of plastics with C‐C backbones have therefore mostly been carried out with preoxidized or thermally pretreated substrates under laboratory conditions (Motta *et al*., 2009; Ojeda *et al*., 2009; Jeyakumar *et al*., 2013; Restrepo‐Flórez *et al*., 2014).

Polyethylene is the most common plastic with a C‐C backbone. Various types of PE have been subjected to biodegradation studies in the last decades (Restrepo‐Flórez *et al*., 2014; Sen and Raut, 2015). Microbial enzymes capable of degrading lignin, a heterogeneous cross‐linked phenolic polymer with oxidizable C‐C bonds found in plant cell walls (Freudenberg and Neish, 1968; Suhas *et al*., 2007), have been reported to be involved in the biodegradation of PE (Restrepo‐Flórez *et al*., 2014; Krueger *et al*., 2015). These include laccases (EC 1.10.3.2.), manganese peroxidase (MnP, EC 1.11.1.13) and lignin peroxidases (LiP, EC 1.11.1.14). As the redox potential required for the breakdown of lignin is considerably lower than for the homogenous C‐C backbone of PE, an efficient degradation of PE by these enzymes can, however, not be expected (Krueger *et al*., 2015).

A thermostable laccase from *Rhodococcus ruber* C208 degraded UV‐irradiated PE films both in culture supernatants and in cell‐free extracts in the presence of copper (Santo *et al*., 2013). As a result of oxidations and polymer chain scissions mainly within the amorphous part of the PE films, an increased amount of carbonyl groups and a reduction in the molecular weight were observed after 2 weeks of incubation with the enzyme at 37°C. A laccase from *Trametes versicolor* also strongly reduced the molecular weight of a PE membrane in the presence of 1‐hydroxybenzotriazole, which mediated the oxidation of non‐phenolic substrates by the enzyme (Fujisawa *et al*., 2001). MnP from the white‐rot fungi *Phanerochaete chrysosporium* ME‐446 and the isolate IZU‐154 have been described as key enzymes for the degradation of a high molecular weight PE membrane (Iiyoshi *et al*., 1998). Surfactants including Tween 80, Tween 20 and CHAPSO have been shown to promote the degradation of PE by the partially purified MnP (Iiyoshi *et al*., 1998; Ehara *et al*., 2000). The genes encoding the most active MnP from IZU‐154 have been identified and further characterized, however only with respect to the oxidation of 2,6‐dimethoxyphenol (Matsubara *et al*., 1996) and the degradation of nylon‐66 (Deguchi *et al*., 1998). *Bacillus cereus* also degraded UV‐irradiated PE associated with a pronounced extracellular production of both laccases and MnP (Sowmya *et al*., 2014). However, the incubation of similarly pretreated PE with a partially purified laccase and a MnP from *Penicillium simplicissimum* resulted only in a negligible weight loss of less than 1% (Sowmya *et al*., 2015). The concentrated culture supernatants of lignocellulose‐degrading *Streptomyces* species containing LiP activity were reported to degrade the PE fraction of a heat‐treated plastic blend (Pometto *et al*., 1992). Similarly, the extracellular LiP and MnP of *Phanerochaete chrysosporium* MTCC‐787 were reported to degrade 70% of a preoxidized high molecular weight PE sample within 15 days of incubation (Mukherjee and Kundu, 2014).

Alkane hydroxylases (AH) of the AlkB family (EC 1.14.15.3) can catalyse the degradation of hydrocarbon oligomers by terminal or subterminal oxidation (Rojo, 2010). A recombinant AH from *Pseudomonas* sp. E4 expressed in *Escherichia coli* BL21 converted 20% of the low molecular weight PE sample to CO_2_ after incubation for 80 days at 37°C (Yoon *et al*., 2012). A recombinant *E. coli* strain simultaneously expressing the complete AH system from *Pseudomonas aeruginosa* E7 including an alkane monooxygenase, rubredoxin and rubredoxin reductase degraded about 30% of this PE sample (Jeon and Kim, 2015).

A purified hydroquinone peroxidase (EC 1.11.1.7) of the lignin‐decolorizing *Azotobacter beijerinckii* HM121 degraded PS, an aromatic thermoplastic with a C‐C backbone (Fig. 1), in a two‐phase system consisting of dichloromethane and water. PS in the organic phase was rapidly converted to small water‐soluble products within 5 min of reaction at 30°C in the presence of hydrogen peroxide and tetramethylhydroquinone (Nakamiya *et al*., 1997). However, this two‐phase process has apparently not been further developed for a recycling process of PS waste. While the degradation of PE and PS by novel bacterial strains isolated from the guts of insect larva has been reported recently, the corresponding enzymes involved have not been identified yet (Yang *et al*., 2014, 2015).

The above‐mentioned studies used only culture supernatants or partially purified enzyme preparations and required long incubation times. For PVC, another important plastic with a C‐C backbone, enzymes directly involved in its degradation have not been reported yet.

The use of whole cells rather than isolated enzymes has been proposed as a potentially better approach for the biodegradation of plastics with C‐C backbones. Recently, the utilization of microbial communities or mixed cultures with defined microbial strains has shown an improved performance in the degradation of PS and PE compared with the use of single microorganisms (Roy *et al*., 2008; Esmaeili *et al*., 2013; Yang *et al*., 2015; Mukherjee *et al*., 2016).

## Enzymatic degradation of PUR

**Figure 5:**
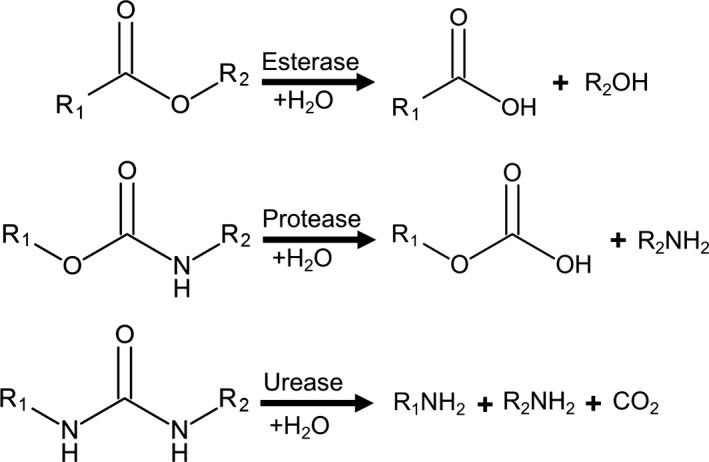
Cleavage of linkages in polyurethane by esterases, proteases and ureases (modified from Phua *et al*., 1987 and Loredo‐Trevino *et al*., 2012).

Polyurethane is a polymer composed of di‐ or polyisocyanate and polyols linked by carbamate (urethane) bonds (Seymour and Kauffman, 1992; Fig. 1). The urethane bond connects the crystalline rigid segments consisting of isocyanate and the chain extender with the amorphous parts composed of a polyester or polyether (Nomura *et al*., 1998; Ruiz *et al*., 1999; Urgun‐Demirtas *et al*., 2007). Depending on the polyols used for the polycondensation reaction, polyether and polyester PUR with different characteristic properties can be manufactured (Seymour and Kauffman, 1992). The presence of aromatic esters and the extent of the crystalline fraction of the polymer are important factors influencing the biodegradation of PUR (Urgun‐Demirtas *et al*., 2007; Cregut *et al*., 2013). PUR can be depolymerized by microbial ureases, esterases and proteases hydrolysing the urethane and ester bonds of the plastic (Howard, 2012a; Loredo‐Trevino *et al*., 2012; Cregut *et al*., 2013; Fig. 5). Enzymes degrading polyester PUR from bacteria (Nakajima‐Kambe *et al*., 1995; Howard and Blake, 1998; Stern and Howard, 2000; Howard *et al*., 2001, 2012b; Schmidt *et al*., 2017; Shah *et al*., 2008a) and fungi (Pathirana and Seal, 1984a,b; Crabbe *et al*., 1994; Russell *et al*., 2011) have been described. It has been postulated that proteases hydrolyse the amide and urethane bonds, while ureases attack the urea linkages (Labow *et al*., 1996; Ruiz *et al*., 1999; Matsumiya *et al*., 2010). Esterases and proteases also hydrolyse the ester bonds in polyester PUR as a major mechanism for its enzymatic depolymerization (Nakajima‐Kambe *et al*., 1999; Tang *et al*., 2001a,b; Howard, 2002). Although a cleavage of urethane bonds in polyether PUR by bacterial (Akutsu‐Shigeno *et al*., 2006) and fungal (Owen *et al*., 1996) hydrolases has been reported, this type of PUR is much more recalcitrant to enzymatic attack compared with polyester PUR (Nakajima‐Kambe *et al*., 1999; Christenson *et al*., 2006). However, a weight loss of a polyether PUR of more than 60% following incubation with fungal isolates has been reported recently without yet a characterization of the enzymes involved (Álvarez‐Barragán *et al*., 2016).

The enzymatic hydrolysis of insoluble PUR polymer is a surface erosion process depending on the efficient adsorption of the biocatalysts on the polymer surface prior to the polymer breakdown (Akutsu *et al*., 1998). Compared with the wild‐type enzyme, an increased yield in degradation products was observed following the incubation of a solid polyester PUR with a polyamidase from *Nocardia farcinica* fused to the hydrophobic polymer binding module of the polyhydroxyalkanoate depolymerase from *Alcaligenes faecalis* (Gamerith *et al*., 2016). In contrast, the enzyme with the fused binding module did not show a better performance than the wild‐type enzyme in the hydrolysis of soluble PUR substrates, confirming the importance of the initial enzyme adsorption process in the degradation of solid PUR.

Two types of PUR esterases with apparent synergistic activities in the degradation of PUR have been reported (Nakajima‐Kambe *et al*., 1995, 1999; Akutsu *et al*., 1998; Howard and Hilliard, 1999). A membrane‐bound esterase of *Delftia acidovorans* was shown to be essential for the adhesion of the microorganism on the polymer surface as well as its initial hydrolysis. A second secreted PUR esterase benefitting from the close vicinity to the substrate catalysed the subsequent depolymerization of PUR, thereby increasing the surface area for further cell adhesion mediated by the membrane‐bound esterase (Nakajima‐Kambe *et al*., 1995, 1999; Cregut *et al*., 2013).

While previously isolated PUR‐degrading enzymes showed only low catalytic efficiencies, whole‐cell catalysis has been suggested as a more promising approach for a biotechnical recycling process of PUR waste materials (Cregut *et al*., 2013). PUR foam particles and fragments, which represent the most abundant PUR waste materials (Seymour and Kauffman, 1992), will be difficult to attack by microbial catalysts in an aqueous recycling system. A mechanical size reduction in the PUR foam and the use of immobilized biomass have been suggested to overcome the low bioavailability of this PUR waste material (Cregut *et al*., 2013). However, an efficient biocatalytic degradation approach of PUR with application potential in a biotechnical recycling process has not yet been demonstrated.

## Enzymatic degradation of PET

Polyethylene terephthalate is a polymer of terephthalic acid and ethylene glycol linked by ester bonds (Webb *et al*., 2013). Owing to its widespread uses in packaging materials, beverage bottles and the textile industry, the global PET production has exceeded 41.6 million tons in 2014 (Research and Markets, 2015). The durability and the resulting low biodegradability of PET are due to the presence of repeating aromatic terephthalate units in its backbone and the corresponding limited mobility of the polymer chains (Marten *et al*., 2003, 2005). The semi‐crystalline PET polymer also contains both amorphous and crystalline fractions with a strong effect on its biodegradability (see Physiochemical properties of synthetic plastics as obstacles for their enzymatic degradation, Fig. 4). At industrial processing conditions such as fibre spinning, injection moulding and film blowing, PET materials with different degrees of crystallinity are obtained as a result of flow‐induced crystallization which determines the final structure and properties of the semi‐crystalline polymer (Lamberti, 2014; Wang *et al*., 2016). PET beverage bottles with a crystallinity of over 30% (Liu *et al*., 2004) and PET fibres up to 40% (Lee *et al*., 2013) are common PET products which pose a high recalcitrance to enzymatic degradation.

At a temperature close to the glass transition temperature (*T*_g_) of PET above 65°C (Alves *et al*., 2002), the amorphous parts of the polymer become flexible and more accessible to an enzymatic attack (Parikh *et al*., 1993; Ronkvist *et al*., 2009). Consequently, the high *T*_g_ of PET requires enzymes with a high stability in this temperature range (Ronkvist *et al*., 2009; Sulaiman *et al*., 2014; Wei *et al*., 2014b; Then *et al*., 2016). Recently, an enzymatic hydrolysis of amorphous PET at a reaction temperature of 30°C has also been reported. However, only very low degradation rates could be observed (Yoshida *et al*., 2016). A number of lipases (Vertommen *et al*., 2005; Eberl *et al*., 2009; Ronkvist *et al*., 2009), esterases (Liebminger *et al*., 2007) and cutinases (Müller *et al*., 2005; Alisch‐Mark *et al*., 2006; Araujo *et al*., 2007; Nimchua *et al*., 2007; Ronkvist *et al*., 2009; Herrero Acero *et al*., 2011; Chen *et al*., 2013; Wei *et al*., 2014c) from fungal and actinomycete species hydrolyse amorphous PET and modify the surface of PET films and fibres (Zimmermann and Billig, 2011). Carboxylesterases from *Bacillus licheniformis*,* Bacillus subtilis* and *Thermobifida fusca* also partially hydrolysed PET fibres and showed a high activity against PET oligomers (Billig *et al*., 2010; Oeser *et al*., 2010; Ribitsch *et al*., 2011; Lülsdorf *et al*., 2015; Barth *et al*., 2016). Lipases display low activity against PET due to their lid structure covering the buried hydrophobic catalytic centre and the resulting limited accessibility for polymeric substrates (Guebitz and Cavaco‐Paulo, 2008; Eberl *et al*., 2009; Zimmermann and Billig, 2011). In contrast, cutinases lacking a lid structure were able to cause significant weight losses from amorphous PET films (Ronkvist *et al*., 2009; Sulaiman *et al*., 2014; Wei *et al*., 2016). Structural analyses of both fungal (Martinez *et al*., 1992; Longhi *et al*., 1997) and bacterial cutinases (Kitadokoro *et al*., 2012; Roth *et al*., 2014; Sulaiman *et al*., 2014; Miyakawa *et al*., 2015) revealed an exposed active site close to the surface of the enzymes, which is essential for the recognition and interaction with polymeric substrates (Guebitz and Cavaco‐Paulo, 2008; Kitadokoro *et al*., 2012).

The thermostable cutinase HiC from *Thermomyces* (formerly *Humicola*) *insolens* is the most active fungal polyester hydrolase reported so far (Ronkvist *et al*., 2009). After a reaction time of 96 h at 70°C, HiC hydrolysed a low crystalline (7%) PET film almost completely, suggesting that the crystalline part of the PET film was also degraded at this reaction temperature. The thermostable bacterial LC‐cutinase hydrolysed approximately 25% of a low crystalline PET film for 24 h at the same reaction temperature (Sulaiman *et al*., 2014). The gene encoding this enzyme is homologous to the polyester hydrolases of *Thermobifida* species (Herrero Acero *et al*., 2011) and has been isolated from a plant compost metagenome (Sulaiman *et al*., 2012). Bivalent metal ions such as Ca^2+^ and Mg^2+^ enhanced the thermostability of several polyester hydrolases from actinomycetes and resulted in an increased hydrolytic activity against PET near the *T*_g_ of PET (Thumarat *et al*., 2012; Kawai *et al*., 2014; Sulaiman *et al*., 2014; Miyakawa *et al*., 2015; Then *et al*., 2015, 2016; Wei *et al*., 2016). By substitution of the metal binding site with a salt bridge or a disulfide bridge, variants of the polyester hydrolase TfCut2 from *Thermobifida fusca* KW3 also readily degraded amorphous PET films at 70°C in the absence of metal ions (Then *et al*., 2015, 2016). The stabilizing effect of phosphate anions at a concentration of up to 1 M (Jensen *et al*., 1995; Park *et al*., 2001) also promoted the activity of these enzymes against PET (Schmidt *et al*., 2016).

**Figure 6:**
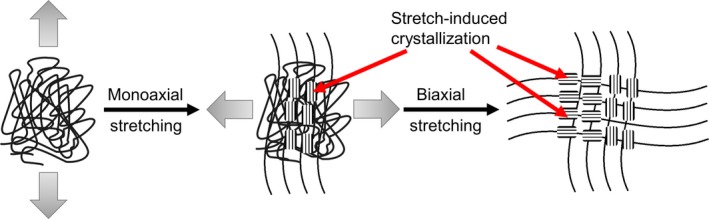
Schematic illustration of the biaxial stretching of an amorphous polymer and the stretch‐induced crystallization (Zhang *et al*., 2016). Due to the small crystallite size below the wavelength of visible light, the resulting material remains transparent after the biaxial stretching (Jariyasakoolroj *et al*., 2015).

Although some polyester hydrolases displayed high activity against amorphous PET materials at reaction temperatures above 50°C, crystalline and biaxially oriented PET was degraded to a much lesser extent at the same reaction conditions (Eberl *et al*., 2009; Ronkvist *et al*., 2009; Wei *et al*., 2016; Gamerith *et al*., 2017; Fig. 6). It is therefore presently not possible to completely degrade PET fibres and beverage bottles with a higher percentage of crystallinity (Liu *et al*., 2004; Lee *et al*., 2013) or biaxially oriented PET within short reaction times with these enzymes (Zhang *et al*., 2004; Zimmermann and Billig, 2011; Carniel *et al*., 2016; Yoshida *et al*., 2016; Gamerith *et al*., 2017).

The enzymatic hydrolysis of PET is also a surface erosion process (Zhang *et al*., 2004; Müller *et al*., 2005; Mueller, 2006; Wei *et al*., 2014a). The hydrophobic nature of PET represents a barrier which hampers the effective adsorption of enzymes to the polymer surface for the hydrolytic reaction (Atthoff and Hilborn, 2007). Unlike enzymes hydrolysing natural polymers such as polyhydroxyalkanoates (Knoll *et al*., 2009) or cellulose (Atthoff and Hilborn, 2007), specific binding domains responsible for substrate adsorption are absent in cutinases (Chen *et al*., 2013; Wei *et al*., 2014c). Their initial adsorption to the surface of PET is presumably mediated by hydrophobic regions surrounding the catalytic site (Herrero Acero *et al*., 2011). By site‐directed mutagenesis of selected amino acids in these regions, variants with enhanced activity against PET could be obtained (Wei, 2011; Herrero Acero *et al*., 2013). The fusion of polymer and cellulose binding domains (Ribitsch *et al*., 2013) or hydrophobins (Ribitsch *et al*., 2015) also enhanced the adsorption of cutinases to the surface of PET and resulted in higher yields of hydrolysis products. A truncation of 71 N‐terminal residues of an esterase from *Clostridium botulinum* exposed a hydrophobic patch, which facilitated its adsorption to PET and improved its hydrolytic activity (Biundo *et al*., 2016).

Selected amino acid residues in close vicinity to the catalytic centre were suggested to be important for the recognition and interaction with the polymeric substrate by various polyester hydrolases (Guebitz and Cavaco‐Paulo, 2008; Kitadokoro *et al*., 2012). By modification of the size and the hydrophobicity of these residues, the hydrolytic activity against PET of a polyester hydrolase from *Fusarium solani* (Araujo *et al*., 2007) and *Thermobifida fusca* (Silva *et al*., 2011) could be increased.

**Figure 7:**
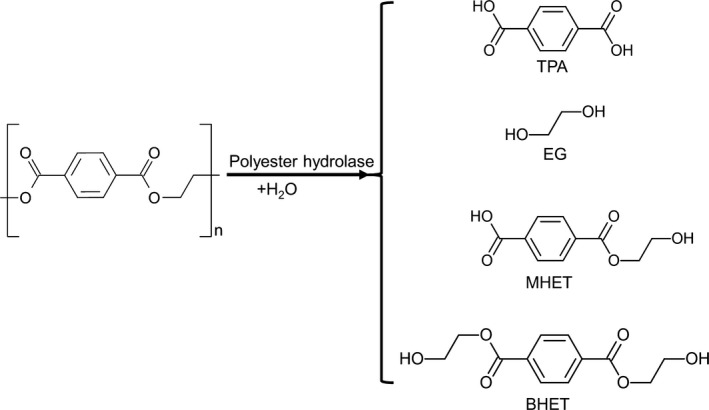
Water‐soluble products obtained from PET films degraded by a polyester hydrolase (Barth *et al*., 2015a). TPA, terephthalic acid; EG, ethylene glycol; MHET, mono‐(2‐hydroxyethyl) terephthalate; BHET, bis‐(2‐hydroxyethyl) terephthalate.

In addition to ethylene glycol, terephthalate, mono‐(2‐hydroxyethyl) terephthalate (MHET) and bis‐(2‐hydroxyethyl) terephthalate (BHET) are the main water‐soluble products obtained by the enzymatic hydrolysis of PET (Vertommen *et al*., 2005; Wei *et al*., 2012; Fig. 7). The polyester hydrolase TfCut2 is strongly inhibited by MHET and BHET (Barth *et al*., 2015a). By performing the hydrolysis of amorphous PET films in an enzyme reactor fitted with an ultrafiltration membrane, the product inhibition could be avoided by the continuous removal of MHET and BHT (Barth *et al*., 2015b). As a result, a 1.7‐fold higher amount of hydrolysis products were obtained from amorphous PET films after a reaction time of 24 h. With a dual enzyme reaction system composed of a polyester hydrolase and the immobilized carboxylesterase TfCa from *Thermobifida fusca* KW3, a twofold higher yield of degradation products could be obtained compared with those without TfCa (Barth *et al*., 2016). In this one‐pot process, TfCa prevented the inhibition of TfCut2 by specifically binding and hydrolysing MHET and BHET in the reaction medium. Similarly, a reaction system composed of the fungal polyester hydrolase HiC and the lipase CalB from *Candida antarctica* also showed a 7.7‐fold increase in the yield of terephthalate obtained due to the concomitant degradation of MHET catalysed by CalB (Carniel *et al*., 2016). A recently described enzyme from *Ideonella sakaiensis* 201‐F6, which catalysed specifically the hydrolysis of MHET (Yoshida *et al*., 2016), could be a further candidate for a one‐pot system to promote PET hydrolysis in an enzyme reactor. The susceptibility of TfCut2 to product inhibition could also be mitigated by modifying a key amino acid residue involved in the interaction with a low molecular weight PET model compound (Wei *et al*., 2016). As a result, a 2.7‐fold higher weight loss of an amorphous PET film was obtained with this variant after a reaction time of 50 h compared with the wild‐type enzyme.

## Prospects for a biocatalytic recycling of recalcitrant plastic waste

The recovery of chemical feedstocks that can be used for the production of virgin polymers in a closed‐loop recycling process is considered as the most sustainable option to solve the plastic waste problem (Al‐Salem *et al*., 2009; Andrady, 2015c). A drastic increase in the amount of plastic waste being recycled instead of discarded will therefore be necessary in the future (World Economic Forum, 2016). Increasing knowledge on microbial enzymes able to degrade petroleum‐based recalcitrant plastics will promote the further development of environmentally friendly plastic recycling processes (Müller *et al*., 2005; Barth *et al*., 2015b, 2016). Although several redox enzymes have been found to contribute to the degradation of PE, a complete biocatalytic degradation of plastics with C‐C backbones has not been demonstrated yet. The identification of suitable enzymes and a better understanding of the degradation mechanism will be necessary before an application of enzymes for a recycling of PE waste could be envisaged. Instead, whole‐cell catalysis with single microorganisms or even microbial communities might provide an alternative strategy for a biotechnical PE recycling.

Synthetic polyesters such as PET and also polyester PUR have been shown to be susceptible to enzymatic degradation by microbial polyester hydrolases. A range of fungal and bacterial enzymes has been described recently able to modify and degrade PET films and fibres. As a proof of principle, the conversion of amorphous PET in an enzyme reactor to its monomers has been demonstrated recently (Barth *et al*., 2015b). Although the crystalline parts of PET can also be degraded by the enzymes, this process is still too slow to be applied for a biocatalytic recycling of PET beverage bottles or textile fibres. Metagenomic approaches have already shown to facilitate the access to novel polyester hydrolases from the environment (Sulaiman *et al*., 2012). Directed evolution strategies can be applied for the identification of mutation hot spots in polyester hydrolases complementing enzyme optimization strategies by semi‐rational re‐design (Thumarat *et al*., 2012; Kawai *et al*., 2014). In the search for improved biocatalysts, high‐throughput screening methods specifically designed to monitor polyester hydrolase activities enable a rapid identification of these enzymes and their variants (Wei *et al*., 2012). The discovery of novel microbial polyester hydrolases and the construction of highly active variants therefore remain a key challenge for the development of a viable biocatalytic recycling process for post‐consumer PET waste.

## Conflict of interest

The authors have declared no conflict of interest.
